# Mitochondrial Replacement Techniques, Scientific Tourism, and the Global Politics of Science

**DOI:** 10.1002/hast.763

**Published:** 2017-09-20

**Authors:** Sarah Chan, César Palacios‐González, María De Jesús Medina Arellano

## Abstract

*The United Kingdom is the first and so far only country to pass explicit legislation allowing for the licensed use of the new reproductive technology known as mitochondrial replacement therapy. The techniques used in this technology may prevent the transmission of mitochondrial DNA diseases, but they are controversial because they involve the manipulation of oocytes or embryos and the transfer of genetic material. Some commentators have even suggested that MRT constitutes germline genome modification. All eyes were on the United Kingdom as the most likely location for the first MRT birth, so it was a shock when, on September 27, 2016, an announcement went out that the first baby to result from use of the intervention had already been born. In New York City, United States‐based scientist John Zhang used maternal spindle transfer (one of the recognized MRT methods) to generate five embryos for a woman carrying oocytes with deleterious mutations of the mitochondrial DNA. Zhang then shipped the only euploid embryo to Mexico, where it was transferred to the mother's uterus. Zhang's team's travel across international borders to carry out experimental procedures represents a form of scientific tourism that has not been properly ethically explored; it can, however, have seriously detrimental effects for developing countries.*

## policy and politics

The United Kingdom is the first and so far only country to pass explicit legislation allowing for the licensed use of a new reproductive technology: mitochondrial replacement therapy.^1^ The techniques used in this technology may prevent the transmission of mitochondrial DNA diseases, but they are controversial because they involve the manipulation of oocytes or embryos and the transfer of genetic material. Some commentators have even suggested that mitochondrial replacement therapy constitutes germline genome modification, a prospect that has long been the subject of ethical concern.^2^

While the ethical issues raised by MRT continue to provoke academic debate, the United Kingdom has already granted the first license, of a two‐step scheme, to Newcastle Fertility Centre; a second license now needs to be granted to a specific patient, which is yet to happen.^3^ Although a 2016 U.S. Institute of Medicine report asserted that mitochondrial replacement is ethically permissible as long as it is limited to male embryos to avoid germline transmission, the U.S. Food and Drug Administration has been barred from even acknowledging the receipt of applications to carry it out.^4^ It appears that this situation will not change under the Trump administration.

Given these antecedents, all eyes were on the United Kingdom as the most likely location for the first MRT birth, so it was a shock to the scientific community and the world at large when, on September 27, 2016, an announcement went out that the first baby to result from use of the intervention had already been born.^5^ In New York City, United States‐based scientist John Zhang used maternal spindle transfer (one of the recognized MRT methods) to generate five embryos for a woman carrying oocytes with deleterious mutations of the mitochondrial DNA. Zhang then shipped the only euploid embryo to Mexico, where it was transferred to the mother's uterus. The baby was born in April 2016 and is apparently doing well.

While this is a happy result for the new family, the consequences of Zhang's team's actions—crossing borders to achieve an early first in this field—will continue to be felt and have implications for health research in Mexico, the reproductive rights of Mexicans, and the global politics of science. Medical tourism, in which patients travel outside the country where they reside to seek medical care, has received much ethical attention; Zhang's team's travel across international borders to carry out experimental procedures represents a form of *scientific tourism* that has not been properly ethically explored. It can, however, have seriously detrimental effects for developing countries. An awareness of the consequences is essential as we continue to contemplate policy in this controversial area.

## Local Adverse Effects

The first and most immediate concern is the potential for local adverse effects on reproductive health and the regulation of research. In Mexico, a country with a strong conservative vein, embryo research and reproductive health are highly contested areas. The Catholic church and extensive lobbying and protests led by conservative organizations such as the National Family Front have had a significant impact on legislators. Since 2007, several state constitutions around the country have been changed to protect human life from the moment of conception (that is, implantation) or fertilization.^6^

Access to reproductive health technologies and scientific research in this area are thus already in a precarious position. In light of current moves to revise and clarify the federal laws governing assisted reproduction and the use of human embryos, the revelation of Zhang's work may be especially damaging.^7^ The negative publicity directed at this event on the international stage and the associated vilification of Mexico as a country with lax regulation provides ammunition to conservative groups that are seeking to make the law more restrictive.^8^

Further, an absence of explicit regulation does not necessarily mean “anything goes” or that there is no will to regulate. In some cases, a deliberate legal lacuna can itself be a form of regulatory compromise;^9^ at other times, regulation is a debate in progress, moving forward as the result of complex negotiation between competing positions with high political capital. Short‐circuiting this process by taking advantage of interim uncertainties threatens to disrupt this delicate balance and foster a regulatory backlash.

It might be argued that scientific tourism can have beneficial effects for local science, if the transfer of training and technology contribute to local capacity building. In this case, however, given that Zhang's team intends only to transfer the embryos in Mexico while carrying out the MRT procedures in the United States, this seems unlikely. Zhang recently stated that, “[f]or now, our nuclear transfer technique is very much like an iPhone that's designed in California and assembled in China.”^10^ This does not indicate any intention to promote development of Mexican science.

## Consequences for the Long‐Term Development of Science

Beyond these immediate consequences, scientific tourism can have wider implications for the long‐term development of science in under‐resourced destination countries. If regulation becomes more restrictive due to scientific tourism, local scientists will be unable to pursue their research in their home country. Given that scientists in these countries often lack sufficient resources to engage in scientific tourism themselves, the net effect will be to block or substantively delay their work altogether, as has happened to the first Mexican scientist to derive an embryonic stem‐cell line in Mexico.^11^ The adverse impact of this could thus be threefold: It contributes to the “brain drain” of developing countries, with associated negative effects in local scientific communities and health resources. It further disadvantages scientists in countries where research already lacks support or is hampered by unclear regulation. And it affects a country's overall scientific competitiveness in the long term. This, obviously, creates a clear problem of global scientific injustice.

The response to Zhang's work also reflects a deeper problem of what we might call “scientific chauvinism,” whereby criteria for scientific practice, regulatory standards, and the terms of public discourse over science are dictated by the dominant scientific community. Deviations, which often fall out along cultural and political boundaries, are automatically classified as unscientific, unethical, or unacceptable.^12^ However, Zhang's work in Mexico does not reflect different *ethical* standards: the moral sensitivity of this area of research and the need for regulation and oversight is recognized in Mexico, even if the response to that need has so far been less than effective. Advisory groups in both the United Kingdom and the United States, along with much of the bioethics literature, have deemed the technique to be ethically acceptable, at least in principle. Zhang's move to Mexico for the embryo transfer process was therefore more a matter of escaping local oversight than going against ethical prescriptions. Should blame for this be attributed solely to the inadequacy of Mexican regulation?

This mode of allocating responsibility for scientists’ conduct reveals a problem in attitudes toward global science and governance. When ethical questions arise about research in developed countries, the assumption is generally that the scientist must have done something wrong, while the regulatory system and scientific culture is only a secondary object of scrutiny. The mainstream coverage of the ethical problems that emerged in relation to Paolo Macchiarini's work on tissue‐engineering transplants, for example, focused principally on his character and actions and the individual roles of others who enabled his actions, rather than on Sweden's scientific culture and its regulatory and governance systems.^13^

When controversial or ethically dubious work is revealed in developing countries such as Mexico, however, the assumption is often that there is something wrong with the system, be it insufficient regulation, inadequate oversight, or inappropriate ethical standards. Most commentators took on face value Zhang's statement that, in Mexico, “there are no rules.”^14^ In fact, Mexico has rules regarding both research oversight and assisted reproduction; indeed, Zhang's team may have violated Mexican federal regulations on medical research.^15^ Focusing solely on Mexico's apparent failure to conform to the standards of ethics and regulation upheld in supposedly more developed countries deflects attention from the responsibilities of scientists and reinforces biased attitudes about global ethical standards and the governance of science. We may draw a comparison here with gene editing and the ethical skepticism expressed toward the Chinese studies published on embryo gene editing. Such attitudes reflect the perception, possibly unjustified, of a “Wild East” with inferior ethical standards and inadequate regulation.^16^

Finally, Zhang's work also illustrates a problem of justice with respect to regulatory capacity. Scientific tourism can impose an unfair oversight burden on countries where ethics and governance structures for these technologies are under development. Researchers who travel to take advantage of an already overloaded system are unjustifiably increasing the burden of local oversight to further their own academic and other interests. If we consider that the scientific brain drain (of human resources) or so‐called biopiracy (of genetic or biological resources) are problems for global scientific justice, then siphoning off oversight resources by engaging in scientific tourism ought equally to be regarded as problematic.

There is, though, one possible positive outcome of this event for Mexico. Spurred by worldwide attention to Zhang's work, a national debate on assisted reproduction that includes scientists, stakeholders, and bioethicists might ensue, leading to an adequate regulatory framework that does not stifle scientific advancement. This possibility is remote at present due to Mexico's political climate, but it is one that we must try to promote.
